# Murine cytomegalovirus reactivation concomitant with acute graft-versus-host disease is controlled by antibodies

**DOI:** 10.1172/jci.insight.149648

**Published:** 2023-03-08

**Authors:** Martina Seefried, Nadine Hundhausen, Irena Kroeger, Maike Büttner-Herold, Petra Hoffmann, Matthias Edinger, Evelyn Ullrich, Friederike Berberich-Siebelt, William J. Britt, Michael Mach, Thomas H. Winkler

**Affiliations:** 1 Department of Biology, Nikolaus-Fiebiger-Center for Molecular Medicine, Friedrich-Alexander-University Erlangen-Nürnberg (FAU), Erlangen, Germany.; 2 Institute of Pathology, University of Würzburg, Würzburg, Germany.; 3 Department of Internal Medicine 5, Hematology and Oncology, University Hospital, Erlangen, Germany.; 4 Department of Nephropathology, Institute of Pathology, FAU, Erlangen, Germany.; 5 Department of Internal Medicine III, Hematology and Oncology, University Hospital, Regensburg, Germany and LIT - Leibniz Institute for Immunotherapy, University Regensburg, Regensburg, Germany.; 6 Experimental Immunology, Department for Children and Adolescents Medicine, University Hospital Frankfurt, Goethe University, Frankfurt am Main, Germany.; 7 Frankfurt Cancer Institute, Goethe University, Frankfurt am Main, Germany.; 8 Department of Pediatrics, University of Alabama School of Medicine, Birmingham, Alabama, USA.; 9 Institute for Clinical and Molecular Virology, University Hospital, Erlangen, Germany.

**Keywords:** Hematology, Immunology, Stem cell transplantation

## Abstract

Reactivation of human cytomegalovirus (HCMV) from latency is a frequent complication following hematopoietic stem cell transplantation (HSCT). The development of acute graft-versus-host disease (GVHD) is a significant risk factor for HCMV disease. Using a murine GVHD model in animals latently infected with murine CMV (MCMV), we studied preventive and therapeutic interventions in this high-risk scenario of HSCT. Mice latently infected with MCMV experienced reactivated MCMV and developed disseminated MCMV infection concomitant with the manifestations of GVHD. Dissemination was accompanied by accelerated mortality. We demonstrate that MCMV reactivation and dissemination was modulated by MCMV-specific antibodies, thus demonstrating in vivo protective activity of antiviral antibodies. However, the efficacy of serum therapy required repetitive doses of high-titer immune serum secondary to the shortened serum half-life of IgG in animals with GVHD. In a complementary approach, treatment of GVHD by adoptive transfer of donor-derived Tregs facilitated production of MCMV-specific antibodies from newly developing donor-derived B cells. Together, our findings strongly suggest that antibodies play a major role in controlling recurrent MCMV infection that follows GVHD, and they argue for reassessing the potential of antibody treatments as well as therapeutic strategies that enhance de novo antibody development against HCMV.

## Graphical abstract

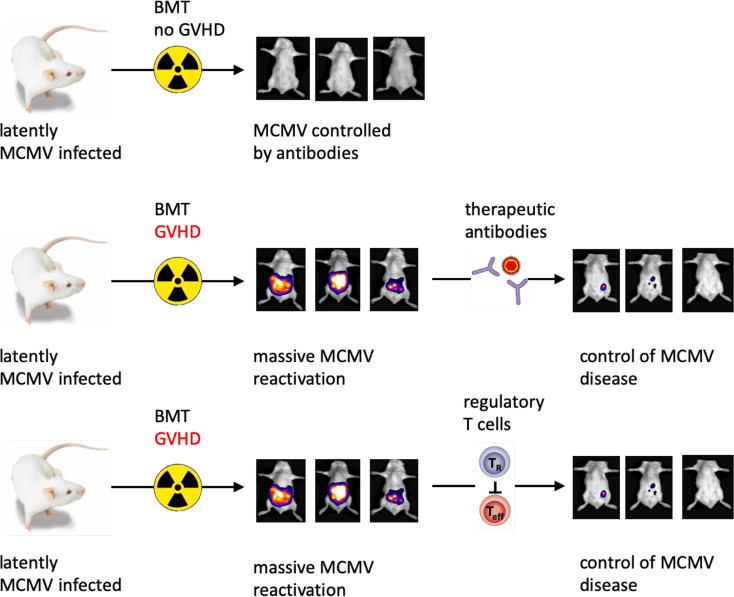

## Introduction

Human cytomegalovirus (HCMV) is an important and ubiquitous human pathogen that is found throughout all geographic areas and socioeconomic groups. Initial infection with HCMV is followed by life-long persistence characterized by episodes of periodic reactivation. Most infections are subclinical in immunocompetent hosts since the virus is controlled by a multilayered and redundant innate and adaptive immune response (1). However, in immunocompromised patients, loss of immune control and dissemination of the virus can result in severe clinical disease. Thus, HCMV remains the most important viral infection after hematopoietic stem cell transplantation (HSCT), especially in high-risk patients (seronegative donor and seropositive recipient), and can lead to life-threatening HCMV disease in ~10% of HSCT recipients (2).

In addition to complications associated with infections, graft-versus-host disease (GVHD) — caused primarily by infusion of mature donor-derived T cells — continues to be a major cause for morbidity and nonrelapse mortality after HSCT (3). Multiple studies identified acute GVHD and its therapy as significant risk factors for HCMV reactivation in seropositive patients with HSCT (4, 5). Moreover, extensive T cell depletion for prevention of GVHD and cases of mismatched or haploidentical HSCT create additional clinical challenges in the management of HCMV infection.

In total, 20%–40% of HCMV-seronegative patients who receive grafts from HCMV-seropositive donors will develop primary HCMV infection (6). Untreated, 50% of patients with HSCT with HCMV reactivation will develop HCMV disease; CMV pneumonia is the most clinically significant manifestation, with a fatality rate of approximately 50% (7). Thus, even in the era of antiviral therapy, CMV infection and subsequent CMV disease still occurs in a significant fraction of patients.

Reconstitution of adaptive and innate immunity plays a pivotal role in the control of HCMV infection after HSCT, and poor postengraftment immune reconstitution represents a major risk factor for the development of severe HCMV infection. A number of studies have identified the presence of antiviral T cell immunity as a crucial factor associated with successful HSCT, and protocols involving adoptive T cell therapy have been successfully implemented in the treatment of transplant recipients (8). In contrast, the impact of the humoral immune response on the clinical outcome of HCMV infections in patients with HSCT remains controversial (9, 10).

Due to the strict species specificity of CMVs, there is a lack of animal models for study of infections with HCMV. However, infection of mice with murine CMV (MCMV) represents a well-characterized and extensively used animal model HCMV infections (11). Reports derived from studies in his model have demonstrated the relevance of antibodies in limiting and controlling viral infection. In immunocompromised mice, several studies showed that primary and recurrent infections are efficiently controlled by transfer of sera from MCMV-immune donors or monoclonal antibodies (12–14). Moreover, Cekinović and colleagues demonstrated that, in MCMV-infected newborn mice, antibody treatment resulted in the clearance of virus from the central nervous system and reduction of virus-related neuropathology (15).

Preclinical as well as clinical studies established an adoptive immunotherapy regimen with CD4^+^FOXP3^+^ Tregs to significantly ameliorate GVHD (reviewed in ref. 16). Nothing is known on the influence of such an adoptive Treg transfer on the development of HCMV-specific antibodies, however. One might envisage a negative role on antibody responses exerted by the regulatory function on T cell help for B cells (17).

In the current study, we have used a mouse model for the analysis of the humoral immune response in an experimental protocol that mimics key aspects of GVHD and MCMV reactivation in recipients of MHC-mismatched allogeneic BM transplantation (allo-BMT). Our results demonstrate that viral reactivation was modulated by MCMV-specific antibodies and that, by repetitive administration of MCMV-immune serum, viral reactivation and disseminated infection could be significantly reduced. Furthermore, adoptive transfer of Tregs allows de novo antibody production by newly generated B cells from the donor while mitigating GVHD. Our studies suggest that antibodies play a major role in controlling recurrent MCMV infection after allo-BMT and suggest reassessing the value of antibody therapy as well as therapeutic strategies that enhance de novo antibody development against HCMV for preventing severe HCMV infection after stem cell transplantation.

## Results

### BALB/c mice latently infected with MCMV demonstrate reactivation of viral infection concomitant with manifestations of GVHD.

**Figure 1:**
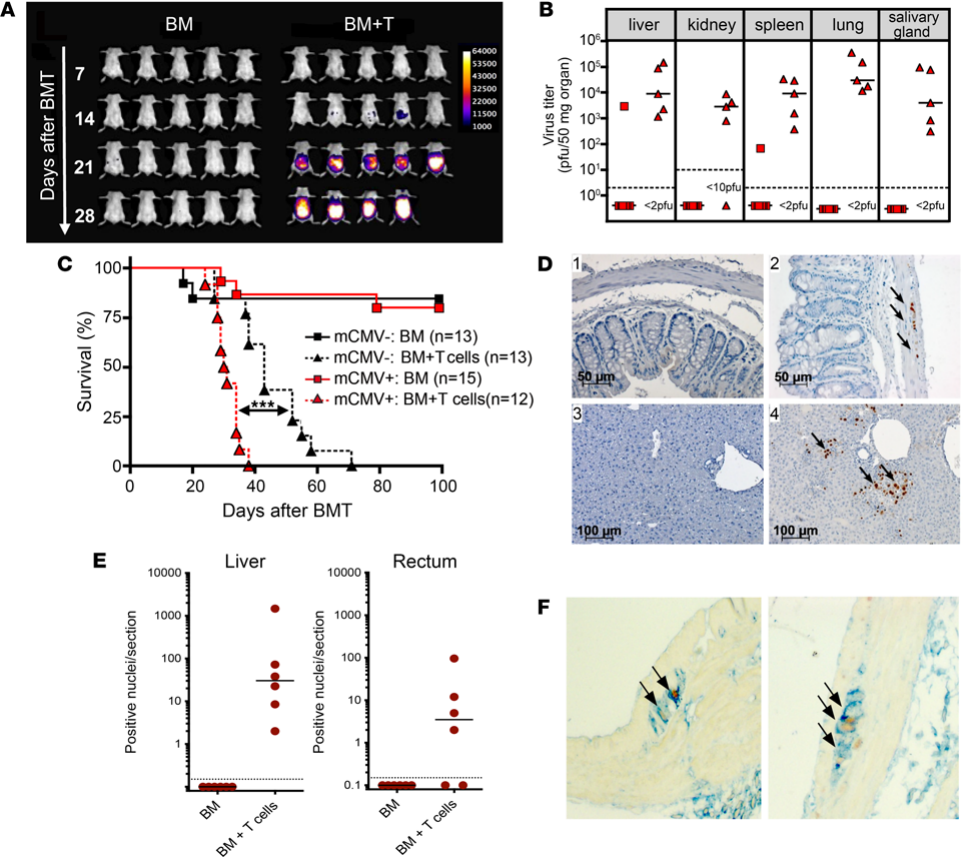
GVHD results in recurrent infection in MCMV-infected BMT recipients. MCMV-infected BALB/c mice were lethally irradiated and received 5 × 10^6^ T cell-depleted bone marrow cells (BM) ± 6 × 10^5^ to 8 × 10^5^ T lymphocytes from C57BL/6 donors. (**A**) Bioluminescence imaging of 5 mice per group at 7, 14, 21, and 28 days after BMT. (**B**) MCMV viral load in mice transplanted with BM (red square) or BM + T cells (red triangle) 25 days after BMT. Median values are depicted as horizontal bars and detection limit as dashed lines. Representative data from 3 experiments. (**C**) Survival curve of 12–15 infected recipients per group transplanted with BM (red squares) or BM + T cells (red triangles). For comparison of survival, 13 uninfected recipients per group were transplanted in parallel with BM (black squares) or BM + T cells (black triangles). MCMV-infected mice that received BM + T cells showed significantly accelerated mortality compared with uninfected recipients receiving BM + T cells. ***P < 0.001; log-rank test. Results of 2 independent experiments were combined. (**D**) IHC stainings of sections from rectum (1 and 2) and liver (3 and 4) from BALB/c infected mice 30 days after transplantation without (1 and 3) or with (2 and 4) GVHD. Immediate-early protein 1 (IE) protein from MCMV was detected in the nucleus of infected cells, as labeled by the arrows. (**E**) Quantification of IE^+^ nuclei in liver and rectum of transplanted mice. (**F**) Double stainings of rectum of GVHD mice for glial fibrillary acidic protein (GFAP) in blue and MCMV IE protein in brown showing colocalization as labelled by the arrows.

Previously, small-animal models for HCMV infections after allogeneic stem cell transplantation have used sublethally irradiated mice and acute infection with MCMV (11, 18). To establish a mouse model for reactivation from latent infection, BALB/c mice were infected with 1 × 10^5^ pfu of a luciferase-expressing MCMV strain (MCMV157luc; ref. 12), and viral replication was monitored in vivo using bioluminescence imaging. In the MCMV157luc strain, the m157 gene is replaced by the firefly luciferase gene. thus destroying the ligand of the Ly49H activation receptor on NK cells in the C57BL/6 strain, resulting in increased virulence (19). When assayed at 25 days postinfection (dpi), viral replication was not detectable in the animals, thus indicating that acute infection had been resolved in these animals (Supplemental Figure 1; supplemental material available online with this article; https://doi.org/10.1172/jci.insight.149648DS1). Four weeks after infection BALB/c mice were lethally irradiated with a single dose of 8 Gy and transplanted with 5 × 10^6^ T cell–depleted BM cells from C57BL/6 donors. In addition, 1 group received purified splenic C57BL/6 T cells for induction of acute GVHD. Transfer of splenic MHC mismatched T cells resulted in a severe GVHD beginning 10–15 days after cell transfer. Using in vivo bioluminescence imaging, viral replication was detectable on day 14 after BMT in T cell recipient mice concomitant with the development of clinically apparent GVHD and increased significantly at later time points in these animals (Figure 1A). In contrast, mice that received BM alone and remained free of signs of GVHD had little to no detectable bioluminescence signal. To correlate the in vivo bioluminescence data with viral load in selected organs, animals were sacrificed on day 25 after transplantation, and infectious virus was quantified in different organs. Mice with GVHD had a high viral burden in all organs, consistent with disseminated virus infection (Figure 1B). In mice without GVHD, the quantity of infectious virus was below the level of detection in almost all mice and in most organs (Figure 1B).

MCMV-infected mice with GVHD exhibited an accelerated mortality compared with uninfected mice with GVHD (*P* < 0.001, Figure 1C), suggesting that viral infection contributed to accelerated mortality in animals with GVHD, as the clinical GVHD scores were comparable in infected and uninfected mice (Supplemental Figure 2). Significant and mostly focal infection in the livers of MCMV-infected mice with GVHD was observed 30 days after transplantation (Figure 1, D and E). In addition, MCMV-infected cells identified by nuclear expression of the immediate early antigen (IE) were readily detectable in the rectum of mice with GVHD (Figure 1, D and E). Interestingly, MCMV infection was detected predominantly in the muscularis propria of the rectum (Figure 1D). Double labeling of sections from the rectums of these mice confirmed a colocalization of MCMV and the glial cell marker glial fibrillary acidic protein (GFAP) in the infected cells (Figure 1F). This finding raised the possibility that MCMV persisted in the ganglia of the enteric nervous system in latently infected mice. MCMV IE^+^ cells were not detectable in matched tissues from any of the infected mice that received only BM and did not develop GVHD (Figure 1, D and E). Previous reports in renal transplant recipients and, more recently, descriptions of tissue from aborted fetuses infected with human HCMV have demonstrated the presence of HCMV in myenteric ganglia from the large intestines (20, 21).

### GVHD after allo-BMT impedes lymphoid reconstitution.

**Figure 2:**
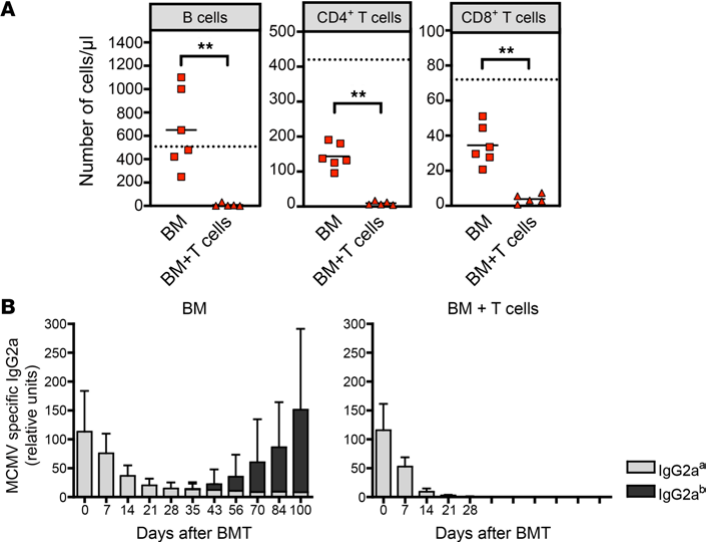
Impact of GVHD on lymphoid immune reconstitution and MCMV-specific antibody kinetics. MCMV-infected BALB/c mice were lethally irradiated and received 5 × 10^6^ T cell–depleted BM cells ± 6 × 10^5^ to 8 × 10^5^ T cells from C57BL/6 donors. (**A**) Flow cytometric analysis of B cells, CD4, and CD8 T cells in peripheral blood. Blood samples were taken from mice transplanted with BM (red square, *n* = 6) or BM + T cells (red triangle, *n* = 5) on day 30 after BMT and stained with fluorochrome conjugated anti-CD19, anti-CD3, anti-CD4, anti-CD8, and anti-CD45.2 antibodies. Mean values are depicted as horizontal bars and mean values of age-matched untreated BALB/c mice (*n* = 4) as dashed lines. **P < 0.01; Mann-Whitney *U* test. Representative data from 3 independent experiments are shown. (**B**) MCMV-specific serum IgG2a titers of mice transplanted with BM (*n* = 7) or BM + T cells (*n* = 4) were analyzed on the days indicated. Detection of IgG2a allotypes a and b allowed the distinction between recipient (IgG2a^a^, gray) or graft (IgG2a^b^, black) origin. Data are shown as mean ± SD.

The adaptive immune system plays a critical role for the clearance of acute MCMV infection in immunocompetent hosts (22). In contrast to immunocompetent C57BL/6 WT mice, immunocompromised Rag1^–/–^ C57BL/6 mice, which lack mature B and T cells, succumb to a MCMV infection with MCMV157luc (12). This is most likely due to the deletion of m157 in this virus strain; this deletion impedes disease control by NK cells that is otherwise observed in C57BL/6 hosts (19). Thus, it was conceivable that a delayed or inadequate reconstitution of lymphoid cells in BMT recipients with GVHD favored the disseminated MCMV infection. Therefore, we analyzed the reconstitution of cells of the adaptive immune system in peripheral blood 30 days after transplantation and the kinetics of MCMV-specific IgG2a titers of recipient (IgG2a^a^) and donor (IgG2a^b^) origin. BALB/c recipients with GVHD had very low to no detectable B cells, CD4 T cells, or CD8 T cells in peripheral blood as compared with transplanted control mice without GVHD (Figure 2A), suggesting that loss of MCMV control in recipient mice with GVHD was caused by profound GVHD-induced immunodeficiency. In agreement with these data, within 3 weeks after transplantation, MCMV-specific IgG2a^a^ (recipient) titers decayed rapidly in animals developing GVHD, and no newly generated antiviral IgG2a antibody responses from B cells of graft origin (IgG2a^b^) were detected (Figure 2B). In contrast, BMT recipients of T cell–depleted BM that did not develop GVHD generated MCMV-specific serum titers from the C57BL/6 BM graft. Interestingly, the loss of detectable virus-specific IgG2a host antibodies coincided with the time of MCMV reactivation (Figure 1A).

### Anti-MCMV antibodies in the transplant recipient protect from MCMV reactivation and disseminated infection.

As shown above, there was incomplete lymphoid reconstitution in mice with GVHD and an accelerated and nearly complete decline of MCMV-specific IgG2a titers. Moreover, the decline of MCMV antibodies correlated with progression of MCMV infection. Previous studies have demonstrated the protective capacity of MCMV-specific antibodies in prophylactic (23, 24) and a more limited number of therapeutic immune serum transfer protocols (12, 14, 15). To evaluate the impact of antibodies on MCMV reactivation and disease in BMT recipients, we used B cell–deficient BALB/c mice (25). Latent viral DNA load had been shown to be comparable in B cell–deficient mice compared with WT mice (23). In addition, we analyzed MCMV infection in B cell–deficient BALB/c mice and found no difference in MCMV infection by bioluminescence imaging (Supplemental Figure 3). These data rule out that absence of antibodies in the primary infection leads to higher numbers of infected cells.

We first analyzed the role of antibodies in the transplant host in the absence of GVHD. To this end, C57BL/6 mice were treated with B cell–depleting anti-CD20 antibodies before infection and monthly thereafter. Latently infected mice were transplanted with syngeneic BM, and MCMV reactivation was measured by bioluminescence on day 8 after transplantation. A significant reactivation of MCMV was observed in anti-CD20–treated mice, and this negatively correlated with significantly less IgG anti-MCMV antibodies in the anti-CD20–treated mice (Supplemental Figure 4). These results show that antibodies in the infected host can significantly ameliorate MCMV reactivation already in a non-GVHD BM transplantation situation, extending the observations of Jonjic et al. (23) to a syngeneic BM transplant setting.

**Figure 3:**
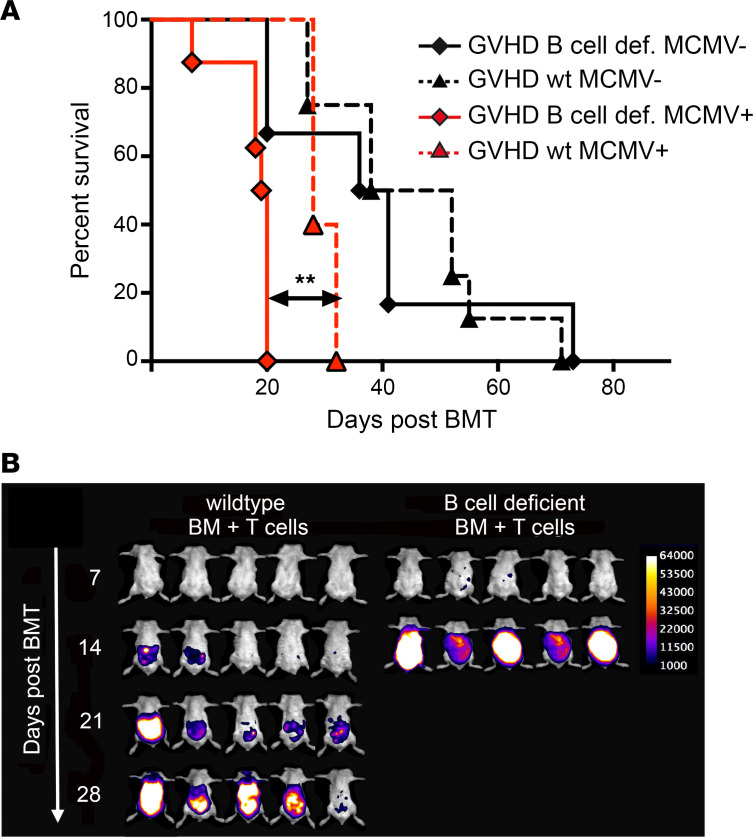
Endogenous recipient antibody titer delayed viral dissemination and prolonged overall survival of recipient mice. Uninfected or MCMV-infected B cell–deficient BALB/c mice were lethally irradiated and received 5 × 10^6^ T cell–depleted BM cells and 8 × 10^5^ T cells from C57BL/6 donors. (**A**) Survival curve of infected (red diamond, *n* = 8) or uninfected (black diamond, *n* = 7) B cell–deficient recipients transplanted with BM + T cells. For comparison, a control group of MCMV-infected (red triangle, *n* = 6) or uninfected (black triangle, *n* = 8) BALB/c WT recipients transplanted with BM + T cells is depicted. **P < 0.01; log-rank test. (**B**) Bioluminescence imaging of 5 infected B cell–deficient mice at 7, 14, 21, and 28 days after BMT. For comparison, a control group of 5 MCMV-infected BALB/c WT recipients transplanted with BM + T cells is depicted. Data presented are representative of 2 independent experiments.

B cell–deficient mice that were infected > 25 days prior to BMT succumbed significantly earlier after the development of GVHD when compared with WT mice (Figure 3A). Three weeks after transplantation, < 10% of B cell–deficient mice were alive as compared with 100% of mice in the control group (Figure 3A). There was no difference in survival secondary to GVHD-mediated mortality in the uninfected control groups, suggesting that the increased mortality of MCMV-infected B cell–deficient mice could not be attributed to divergent courses of GVHD in the presence or absence of B cells (Figure 3A). Bioluminescence data of infected B cell–deficient mice revealed a more rapid dissemination of MCMV in the B cell–deficient group compared with WT recipients with GVHD (Figure 3B). These results suggest that antibodies present in the WT but not in B cell–deficient recipients could modify the course of MCMV infection and prolong the survival of recipient mice.

### Therapeutic transfer of immune serum protects from recurrent MCMV infection.

**Figure 4:**
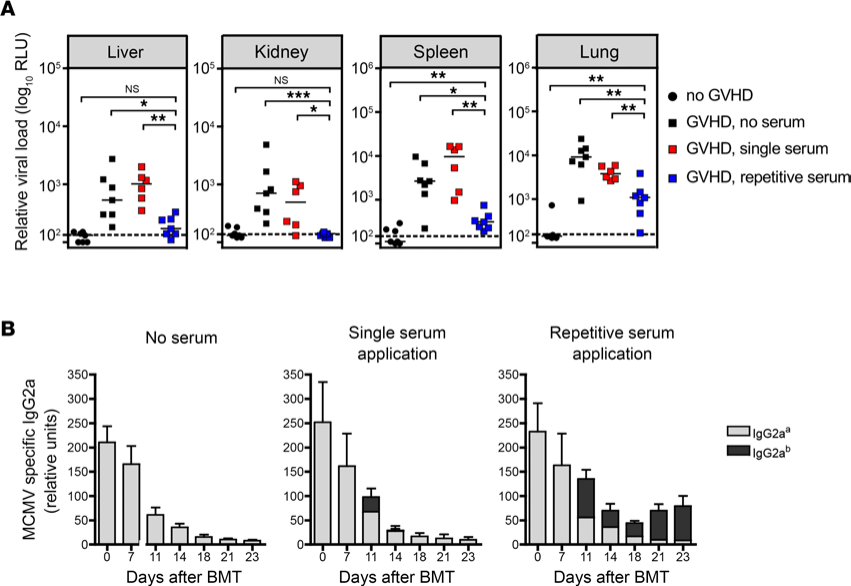
Repetitive treatment of infected donors with immune serum protects from a recurrent MCMV infection. MCMV-infected BALB/c mice were lethally irradiated and received 5 × 10^6^ T cell–depleted BM cells ± 8 × 10^5^ T cells from C57BL/6 donors. Eight days after BMT, 6–7 recipients transplanted with T cells received either a single application of immune serum (200 μL), repetitive treatment with immune serum (200 μL on day 8 postirradiation and 100 μL every second day) or no serum. (**A**) Relative viral load in untreated mice (black squares), in mice after a single treatment with serum (red squares), or after repetitive treatments with sera (blue squares). Mice transplanted only with BM (black circles) served as controls. Median values are depicted as horizontal bars and mean values of uninfected BALB/c (*n* = 3) as dashed lines. *P < 0.05, **P < 0.01, ***P < 0.001; Mann-Whitney *U* test. (**B**) MCMV-specific serum IgG2a titers were analyzed at different time points after BMT. By using the IgG2a allotypes a and b, a distinction between recipient (IgG2a^a^, gray) or immune serum (IgG2a^b^, black) origin was possible. Data are shown as mean ± SD. Representative data from 2 independent experiments are shown.

Thus far, our results have shown that MCMV-specific antibodies might have a protective effect, even in the presence of GVHD. We therefore determined whether this protective effect of MCMV-specific antibodies could be corroborated by passively transferring MCMV antibodies into MCMV-infected animals in the posttransplant period. Previously, we reported that a single dose of immune serum can completely control MCMV infection in infected C57BL/6 Rag1^–/–^ mice for at least 14 days when given at day 3 of infection (12). In a modification of this protocol, we induced lethal GVHD in latently MCMV-infected BALB/c recipients and treated them either with a single dose of C57BL/6-derived (IgG2a^b^) immune serum (200 μL) on day 8 after BMT or with repetitive injections of immune serum (200 μL on day 8 after BMT and 100 μL every second day thereafter). On day 23 after transplant, recipients were sacrificed and viral load was determined in selected organs (Figure 4A). Mice receiving repetitive serum application showed a significantly lower viral burden as compared with untreated mice that reached a 77%–99% reduction of viral load in target organs. Importantly, the viral load in organs of mice given a single serum application remained comparable with mice that did not receive serum. Analysis of MCMV-specific serum IgG2a^a^ titers revealed only a short-term increase of MCMV-specific IgG2a after single serum transfer, whereas repetitive application of immune serum maintained an IgG2a^a^ titer at a constant level that correlated with protection from virus infection (Figure 4B).

### Reduced absorption and shortened half-life of IgG in mice with GVHD.

**Figure 5:**
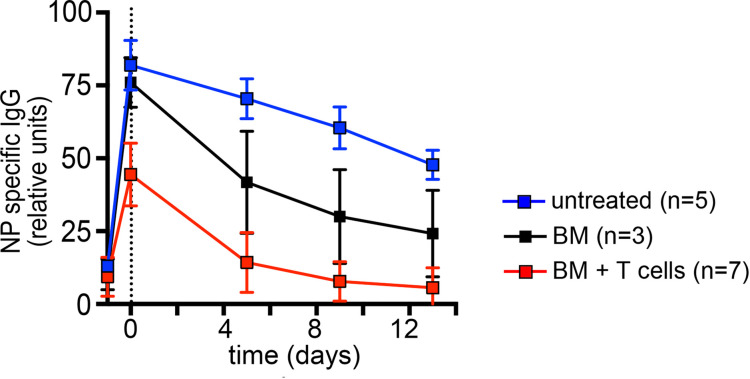
Reduced serum half-life of serum IgG in mice with GVHD. Noninfected BALB/c mice were lethally irradiated and received 5 × 10^6^ T cell–depleted BM cells ± 8 × 10^5^ T cells from C57BL/6 donors. Fourteen days after BMT, recipients received anti–NIP-specific serum. Age-matched nontransplanted BALB/c mice injected with anti–NIP-specific serum served as controls. NIP-specific serum IgG titers were assessed at the indicated time points. Data are shown as mean ± SD. Day of serum injection is indicated by a vertical dotted line. Representative data from 2 independent experiments are depicted.

The finding that a single application of immune serum leads only to a short-term increase of the MCMV-specific IgG2a titer raised the possibility that IgG homeostasis could be altered in recipient mice with GVHD. To exclude the possibility that the observed deficiency of MCMV antibodies in recipient mice with GVHD was secondary to binding to MCMV antigens in the circulation or in the tissue, we injected uninfected BALB/c mice 14 days after BMT with serum containing antibodies reactive against the hapten 4-Hydroxy-3-Nitrophenyl Acetyl (NP), and we analyzed the serum kinetics of IgG anti-NP antibodies after transfer (Figure 5 and Table 1). Untreated and BM-transplanted mice exhibited an efficient absorption of IgG from the peritoneum to the bloodstream, whereas the efficiency IgG absorption following i.p. injection of mice with GVHD was reduced by nearly 50% (Table 1). Moreover, the half-life of IgG in mice with GVHD was reduced to 3 days, a more than 6-fold reduction when compared with untreated control mice and a 3-fold reduction compared with mice following BMT but without GVHD (Table 1). These results revealed that IgG levels were lower in animals with GVHD secondary to diminished absorption of IgG and, more importantly, an accelerated loss of serum antibodies in mice with GVHD. These findings may have important implications for the prophylaxis and/or therapy of HCMV in clinical HSCT.

### Therapeutic intervention by adoptive transfer of Tregs ameliorates MCMV viral burden.

**Figure 6:**
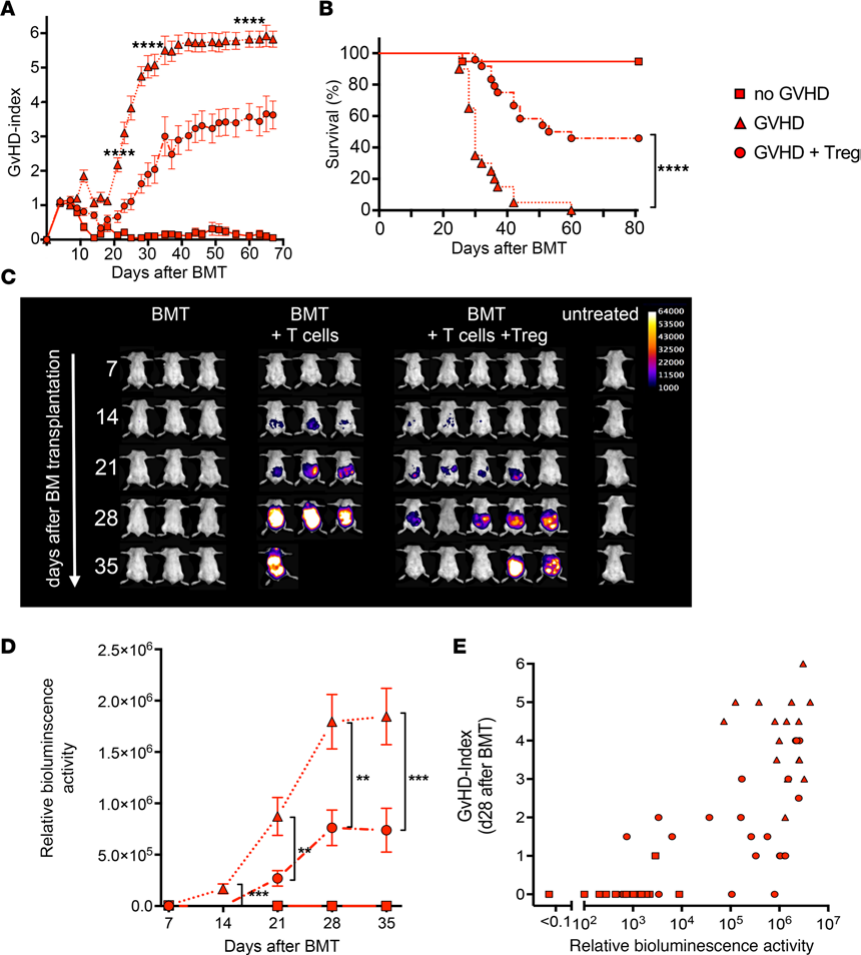
Therapeutic intervention by adoptive transfer of Tregs ameliorates MCMV reactivation. MCMV-infected BALB/c mice were lethally irradiated and received 5 × 10^6^ T cell–depleted BM cells only (squares), coinjected with 8 × 10^5^ T lymphocytes (triangles), or coinjected with 8 × 10^5^ T lymphocytes and 1.2 × 10^6^ CD25^+^ Tregs (circles) from C57BL/6 donors. (**A**) GVHD index over a period of 67 days after BMT. Data collected from 3 independent experiments: BM, *n* = 10; BM+ T_eff_, *n* = 13; and BM + T_eff_+ Treg, *n* = 16. Data are shown as mean ± SEM. ****P < 0.0001; Mann-Whitney *U* test. (**B**) Survival curve of infected recipients over a period of 81 days after BMT. Data collected from 3 independent experiments: BM, *n* = 19; BM+ T_eff_, *n* = 20; and BM + T_eff_+ Treg, *n* = 24. ****P < 0.0001; log-rank test. (**C**) Bioluminescence imaging of 3–5 mice per group at 7, 14, 21, 28, and 35 days after BMT. (**D**) Quantitation of bioluminescence imaging (whole-body bioluminescence). Data collected from 3 independent experiments: BM, *n* = 20; BM+ T_eff_, *n* = 20; and BM + T_eff_+ Treg, *n* = 24. Data are shown as mean ± SEM. **P < 0.01 ***P < 0.001; Mann Whitney *U* test. (**E**) Correlation of GVHD index and viral load day 28 after BMT. BM, *n* = 20; BM+ T_eff_, *n* = 20; and BM + T_eff_+ Treg, *n* = 24. *r* = 0.72, *P* < 0.0001, Spearman’s correlation test.

Thus far, our data have demonstrated a therapeutic value of transfer of MCMV-specific antibodies in preventing and treating disseminated MCMV during GVHD. Since experimental data have shown a clear benefit of the transfer of CD4^+^CD25^+^ Tregs in the prevention and treatment of GVHD in mouse models (26, 27) and in human allograft recipients (28), we analyzed the impact of Treg suppression of GVHD on MCMV reactivation. To this end, we added donor-derived CD4^+^CD25^+^Foxp3^+^ Tregs to the transplantation protocol and studied their influence on GVHD and MCMV reactivation. First, we defined a dose of Tregs that could significantly reduce GVHD and prolong lifespan in uninfected BALB/c recipients (Supplemental Figure 5) (26). Transfer of the same number of Tregs into MCMV-infected BALB/c mice led to a significantly reduced GHVD index (Figure 6A) and a highly significant increase in survival of the animals (Figure 6B). When we analyzed MCMV reactivation in the Treg-treated mice, we observed a lower level of MCMV reactivation in Treg-treated mice that was significantly different from that seen in nontreated GVHD mice at all analyzed time points after transplantation (Figure 6, C and D). Since the reduction of MCMV reactivation was variable in the Treg-treated mice (Figure 6C), we correlated the GVHD index in the individual mice with MCMV virus load as measured by whole body bioluminescence. A significant correlation was observed in a large series of mice analyzed 28 days after transplantation (Figure 6E), suggesting a direct or indirect influence of GVHD suppression on the control of MCMV dissemination. The correlation remained significant when we only analyzed Treg-treated mice (*r* = 0.65, Supplemental Figure 6), further strengthening the notion that the ongoing GVHD reaction is responsible for a relative immunodeficiency and concomitant viral reactivation. Importantly, a large fraction of mice (~45%, Figure 6B) controlled both GVHD as well as MCMV infection and survived longer than 80 days after transplantation.

### Reconstitution of humoral immunity against MCMV during GVHD controlled by Tregs.

**Figure 7:**
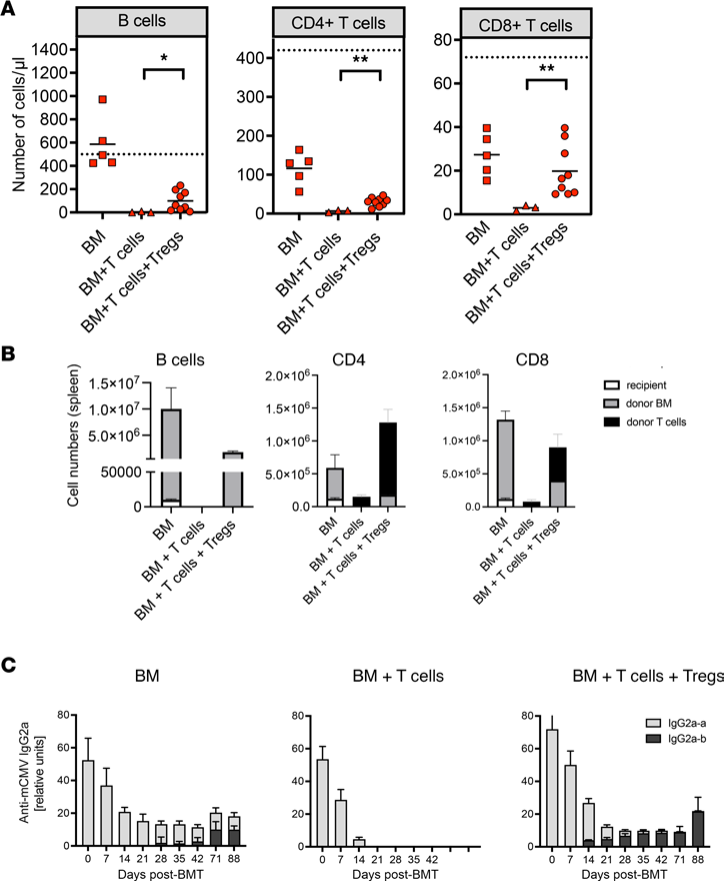
Therapeutic intervention by adoptive transfer of Tregs permits MCMV-specific antibody development de novo. MCMV-infected BALB/c mice were lethally irradiated and received 5 × 10^6^ T cell–depleted BM cells only (squares), coinjected with 8 × 10^5^ T lymphocytes (triangles) or coinjected with 8 × 10^5^ T lymphocytes and 1.2 × 10^6^ CD25^+^ Tregs (circles) from C57BL/6 donors. (**A**) Flow cytometric analysis of B cells, CD4, and CD8 T cells in peripheral blood 30 days after BMT. Mean values are depicted as horizontal bars and mean values of age-matched untreated BALB/c mice (*n* = 4) as dashed lines. *P < 0.05, **P < 0.01; Mann-Whitney *U* test. (**B**) Absolute cell numbers and origin of B cells, CD4, and CD8 T cells 30 days after BMT in recipient spleen. BM cells were from CD45.1 congenic C57BL/6 mice, and splenic donor T cells were from CD45.2 C57BL/6 mice; details of transplantation can be found in Supplemental Figure 7. Data are shown as mean ± SEM cell numbers per spleen. (**C**) MCMV-specific serum IgG2a titers of mice transplanted with BM (*n* = 7), BM + T cells (*n* = 4), or BM + T cells + Tregs (*n* = 4) were analyzed on the days indicated. Detection of IgG2a allotypes a and b allowed the distinction between recipient (IgG2a^a^, gray) or graft (IgG2a^b^, black) origin. Data are shown as mean ± SEM. Two independent experiments with 5–9 individuals/group are shown.

Since GVHD completely abrogated the antibody response against MCMV (Figure 2), we next determined whether Treg treatment leads to the development of anti-viral antibodies in the host and whether these antibodies were derived from de novo B cell responses. In mice treated with Tregs, a normalization of the B cell as well as the CD4 and CD8 T cell compartments in the peripheral blood were observed as compared with nontreated mice with GVHD (Figure 7A), although B cell and CD4 T cell numbers remained lower than those in mice without GVHD after BMT. To analyze the origin of T and B cell populations in more detail, we transplanted T cell–depleted CD45.1 BM cells together with CD45.2 effector T cells with or without CD45.2 Tregs (Supplemental Figure 7). The contributions of recipient-derived (H-2K^d^) versus donor BM–derived (H-2K^b^ CD45.1) or donor spleen–derived (H-2K^b^ CD45.2) B and T cells in recipient spleens 28 days after transplantation is shown in Figure 7B. Essentially all B cells reconstituting in mice under Treg protection were derived from donor BM. After BM transplantation without GVHD, a minor fraction of B cells was derived from the host, suggesting that some remaining radioresistant cells persisted in this setting. Interestingly, a major fraction of CD4 T cells in the spleens of mice reconstituting the lymphocyte compartment in the presence of donor Tregs was derived from transferred splenic donor CD4 T cells rather than from BM. This CD4^+^ T cell compartment, however, also comprises expanded donor Tregs, which we cannot distinguish with our method. The CD8 compartment was equally reconstituted from the cotransplanted splenocyte population and de novo–generated T cells from the donor BM.

Most importantly, when we looked at the development of MCMV-specific antibodies, we observed significant MCMV-specific antibody levels developing concomitantly with the control of GVHD by Tregs in the transplanted mice. These antibodies were entirely derived from donor B cells, as shown by allotype-specific reagents (Figure 7C), presumably generated de novo with CD4 T cell help restricted to the MHC class II allele I-A^b^. We conclude that Treg treatment facilitated lymphocyte reconstitution to an extent that allowed development of a de novo virus-specific antibody responses against MCMV.

Finally, we wanted to analyze whether the de novo–generated MCMV-specific antibodies are essential for control of MCMV after Treg transfer. To this end, we used BM from B cell–deficient C57BL/6 mice for transplantation. The results of this experiment revealed that the absence of development of MCMV-specific IgG from the donor BM after Treg transfer does not alter the 2–3 log reduction of MCMV load in spleen, liver, or lung significantly (Supplemental Figure 8), suggesting redundancy of cellular and humoral immune reconstitution.

## Discussion

Our results provide insights into the association between acute GVHD and HCMV reactivation in patients after BMT and HSCT (29, 30). Latent MCMV reactivation after BMT in this murine model was only observed in recipients with GVHD (induced by cotransplanted alloreactive T cells). In recipients with GVHD, reactivation leads to virus dissemination and high levels of virus replication in multiple organs. Antibodies were shown to modulate virus dissemination and disease following MCMV reactivation and, in addition, could effectively control infection even in the setting of an ongoing GVHD disease. Furthermore, adoptive transfer of Tregs led to a reduction in the severity of GVHD in murine BM recipients, facilitated lymphoid reconstitution that was associated with control of MCMV reactivation and dissemination, and extended survival of transplanted animals. Lastly, transfer of Tregs was associated with a de novo humoral immune response against reactivating MCMV, thus providing an antiviral antibody response in MCMV-infected recipients.

In our experimental model of MCMV reactivation following HSCT, we addressed 3 major risk factors for HCMV reactivation after HSCT: (a) myeloablative conditioning regimen, (b) HCMV-seropositive recipient transplanted with BM from a seronegative donor (R+/D-), and (c) acute GVHD. Within 14 days after BMT, we observed MCMV infection in latently infected mice, and it occurred concomitantly with the manifestations of GVHD. Moreover, MCMV infection progressed to a fulminant disseminated infection that resulted in significantly accelerated overall mortality in this experimental group. In contrast, infected recipients without GVHD exhibited neither significant virus reactivation and dissemination nor enhanced mortality. This finding clearly points to a causal connection in the murine model between the development of GVHD and severe recurrent MCMV infection with increased mortality.

Immune suppression as well as development of an inflammatory milieu have been well described as key factors required for MCMV reactivation and subsequent disease. In particular, allogeneic T cell responses are characterized by a proinflammatory cytokine milieu. Recent studies in allogeneic and syngeneic transplantation models demonstrated that reactivation of MCMV could be induced in allogeneic transplantation of skin or kidneys (31, 32). TNF-α represents a major mediator of allogeneic responses, and reactivation of latent MCMV can be induced in vivo by treatment with TNF-α (33). TNF-α activates NF-κB and thereby induces the transcription of viral IE genes, a key regulatory checkpoint in the replicative cycle of MCMV (32). Since TNF-α is significantly upregulated in our model of GVHD induction (27), it is possibly part of the molecular network resulting in fulminant MCMV reactivation that is amendable for therapeutic interventions.

Although MCMV reactivation can be induced by sublethal γ-irradiation, additional immune suppressive treatment of the recipient further increases the level of viral reactivation (23, 34). GVHD affects the structural integrity and function of thymus, lymph nodes, and BM and causes lymphoid hypoplasia and dysfunction in the recipient (35, 36). Severe lymphopenia and lack of lymphoid reconstitution can be observed during GVHD, and these findings were confirmed in our GVHD model. In clinical HSCT it is often impossible to quantify the relative contribution of GVHD and the pharmacologic immunosuppression employed for GVHD-prophylaxis and/or therapy for HCMV reactivation. In our model, we have shown that the GVHD-induced loss of lymphoid tissue and function contributes to MCMV reactivation, even in the absence of pharmacologic immunosuppression.

Our data clearly demonstrate a pivotal role of humoral immunity in controlling a recurrent MCMV infection in BM recipients. It, thus, confirms in an animal model our previous studies showing that, in patients with BMT, the titer of antiviral antibodies is correlated with survival (37). Latently infected BALB/c recipients with GVHD showed a steady loss of MCMV-specific serum antibodies, which became undetectable as early as 3 weeks after BMT. In parallel with the decline of virus-specific antibodies, we observed a dramatic increase in the overall viral burden in multiple organs, consistent with virus dissemination. In this model, the reduction in the level of virus-specific antibodies was related to a decreased half-life of preformed antibodies and the lack of newly generated antiviral antibody responses in infected animals with GVHD. The dramatically increased levels of infection and higher mortality rates in B cell–deficient BMT recipients that lacked preformed virus-specific antibodies further demonstrated a critical role of antiviral humoral immunity in the control of MCMV following BMT. The clinical relevance of this finding in this murine model is that, in the case of R+D- HSCT, booster vaccinations to specifically enhance HCMV-specific antibodies in the recipient might reduce the risk of HCMV reactivation, particularly during early acute GVHD. With the development of new highly immunogenic mRNA vaccines against HCMV (38), such preemptive interventions might become available in the future.

Previous studies have demonstrated the protective activity of antibodies following the therapeutic transfer of immune serum or monoclonal antibodies in immunodeficient recipients (12, 14, 15). In this report, we demonstrate the protective capacity of virus-specific antibodies in the context of allo-BMT. Similar results were described recently in a different, more aggressive GVHD model that was also focused on the importance of viral strain strain-specific antibodies (39). Interestingly, a single treatment with immune serum was not sufficient to significantly reduce the viral load in the model described in the current report. This lack of protection was unexpected because a similar serum transfer was effective in animal models of MCMV infection that did not include BMT (12, 14). We were able to attribute this low level of protective activity of the immune serum to a reduced efficiency of antibody absorption and a decreased half-life of serum IgG. More importantly, however, we now show that GVHD dramatically shortens the half-life of serum IgG by unknown mechanisms that might relate to the recently observed severe damage, structural change, and dysfunction of the vasculature during GVHD (40). We speculate that the dysfunction of endothelial cells results in disturbance of the IgG homeostasis mediated by the endothelial FcRn (41). In consequence, repetitive serum treatment was required to reach protective serum titers, a finding that may be highly relevant for translation into improved care for HSCT recipients. It is possible to engineer the Fc portion of antibodies to extend the half-live of antibodies to provide longer protection and still allow neutralization as well as cellular effector functions (42).

Our experimental model allowed the analysis of Tregs as an effective measure for prevention and treatment of GVHD (26, 28) in a setting of MCMV reactivation in this model. Previously published data focus on the cellular response after Treg therapy and, most importantly, do not analyze reactivation of MCMV from host tissue as is frequent in the R+D- situation (43, 44). Our data indicate that combined application of Tregs and T cells did not completely control reactivation of MCMV but rather significantly slowed viral dissemination, despite the development of CD8 T cells in the recipient animals. However, Treg treatment facilitated the de novo development of MCMV-specific IgG antibodies. Our analysis of the contribution of specific antibodies from donor or host B lymphocytes clearly shows that these antibodies were produced by B cells regenerated from donor BM stem cells. Despite active suppression of GVHD by Tregs, CD4^+^ T cell–dependent antibody responses against MCMV (12) can be generated even with relatively low numbers of reconstituted donor B lymphocytes and T lymphocytes being present in the secondary immune organs. These findings may well be important in other infections that cause a significant risk in patients after HSCT. Nonetheless, our experiment using B cell–deficient BM led us to conclude that these MCMV-specific antibodies were not the essential mechanism to control MCMV dissemination and that other cellular effector mechanisms also contribute. In terms of potential clinical translation, our data provide a rationale for therapeutic interventions in R+D- patients that concentrate on rapid B cell regeneration and in vitro priming and expansion of HCMV-specific CD4^+^ donor T cells (45).

## Methods

### Mice.

C57BL/6 (H-2b) and BALB/c (H-2d) mice were obtained from Charles River Laboratories (Sulzfeld, Germany). C57BL/6 Rag1^–/–^ mice were originally obtained from Irmgard Förster (University Munich, Germany). BALB/c mb1-MerCreMer mice (Cd79a^tm2[cre/Esr1*]Reth^, MGI:4414764) were a gift from Siegfried Weiss (Helmholtz Centre for Infection Research, Braunschweig, Germany) and were used as homozygous mutant mice in which B cells are completely absent due to the absence of CD79a/Igα (25). C57BL/6 mb1-cre mice (Cd79a^tm1[cre]Reth^, MGI:3687451) were also used as homozygous mutant mice as BM donor mice in 1 experiment. Mice were used at 8–14 weeks of age and were maintained in a specific pathogen–free environment in the animal facility at the Franz-Penzoldt-Zentrum (University Erlangen).

### Virus.

Recombinant virus MCMV157luc has been described previously (12). The virus was propagated in mouse embryo fibroblasts (MEF) and purified as described (46). For inoculation, the virus titer was determined by end-point titration using indirect immunofluorescence staining as described previously (12). In order to generate latently infected recipients for BMT, BALB/c WT and BALB/c mb1-MerCreMer mice were infected i.p. with 1 × 10^5^ plaque-forming units (pfu) of MCMV157luc. To ensure successful primary infection, viral infection was monitored using in vivo bioluminescence imaging. Mice with no detectable virus infection were excluded from further analysis.

### MCMV immune serum and NIP-BSA–specific serum pools.

MCMV immune serum pools were collected from C57BL/6 mice infected i.p. with 1 × 10^6^ pfu of MCMV157luc for 90 days. Protective capacity of immune serum pools was determined by immune serum transfer experiments in infected C57BL/6 Rag1^–/–^ mice (14). For the generation of 4-hydroxy-3-iodo-5-nitrophenylacetic acid bovine serum albumin (NIP-BSA)-–specific serum, C57BL/6 mice were immunized 3 times with 50 μg NIP-BSA in Imject Alum Adjuvant (Thermo Fisher Scientific) at intervals of 4 weeks. Four weeks after the last immunization, serum was collected and combined to NIP-BSA specific serum pools.

### Detection of antigen-specific immunoglobulins by ELISA.

Sera from experimental mice were analyzed by ELISA to measure MCMV specific IgG2a antibody titers. ELISA plates were coated with lysate of MCMV157luc-infected MEFs (MOI: 0.02 for 72 hours) at a concentration of 7 μg/mL. Sera were applied as serial dilutions of 1:2 in 2% FCS and 0.05% Tween 20 (Sigma-Aldrich) in PBS and compared with 1:2 dilution series of control sera from MCMV157luc-infected C57BL/6 (IgG2a^a^) and BALB/c (IgG2a^b^) mice. These control sera were equilibrated for IgG2a titers and allotted with a value of 100 relative units (RU) for relative quantitation. Detection of allotype-specific MCMV-specific IgG2a was achieved by the use of IgG2a^a^- and IgG2a^b^-specific biotinylated secondary Abs (clones 8.3 and 5.7 respectively, BD Biosciences) and HRP-coupled streptavidin (GE Healthcare). For the detection of heteroclytic NP-specific antibodies, ELISA plates were coated with 10 μg/mL NP13-Ova (Biosearch Technologies). Sera were applied as serial dilutions and compared with dilution series of control sera from NIP-BSA immunized C57BL/6 mice. Detection of NP-specific IgG was achieved by the use of a HRP-coupled Fcγ-specific secondary antibody (catalog 115-035-164, Dianova).

### Flow cytometric analysis.

Peripheral blood samples were collected in heparinized tubes (Greiner Bio-One). In total, 50 μL of whole blood was incubated with 2% FCS and 2 mM EDTA in PBS containing the following staining antibodies: CD3-PerCP-eF710 (17A2, eBioscience), CD4-FITC (GK1.5, BD Biosciences), CD8-PE (53-6.7, BD Biosciences), CD19-FITC (1D3, BD Biosciences), CD19-PE (1D3, BD Biosciences), and CD45.2-APC (104, eBiosciences). For lysis of RBCs, 450 μL of 1× BD FACS Lysing Solution (BD Biosciences) was added. Samples were analyzed using a BD FACSCalibur (BD Biosciences) and FlowJo software. Graded numbers of ProCOUNT counting beads (BD Biosciences) were added to the blood samples to enumerate absolute cell numbers per μL. The gating strategy is described in Supplemental Figure 9.

### In vivo bioluminescence imaging.

For noninvasive imaging analysis, mice were injected i.p. with 0.5 mg D-Luciferin (PJK GmbH) 2 minutes prior to imaging. The mice were then anesthetized using isoflurane and placed in a supine position in a light-sealed chamber. Luciferase activity was recorded over a 200-second integration period by a cooled CCD camera system (C4742-98, Hamamatsu Photonics). Photons emitted from various regions of mouse were acquired using SimplPCI Software (Compix), and images were processed in ImageJ (NIH). Bioluminescence images were represented as pseudocolor images overlaid on gray-scale photographic images of the mice using Adobe Photoshop Software (Adobe Systems).

### Determination of viral load in organs.

Infectious virus present in organs was quantitated by the virus plaque (PFU) assay. Briefly, snap-frozen organs were thawed, weighed, and homogenized by passage through a 100 μm cell strainer (BD Biosciences). Serial 1:10 dilutions of organ homogenates were plated in quadruplicate on permissive MEF monolayers in 48-well plates under condition of centrifugal enhancement (46). Plates were incubated for 3 hours, washed once with PBS, and incubated with culture medium. Five days later, virus titer was determined. Detection limit was 2 to 10 pfu/50 mg organ. Determination of viral load by use of organ luciferase activity was performed as described previously (12).

### BM transplantation, GVHD model, and B cell depletion.

Naive or MCMV-infected female recipient mice were lethally irradiated (800 cGy) and injected i.v. with 5 × 10^6^ T cell–depleted BM cells (CD90.2 MicroBeads, Miltenyi Biotec) from C57BL/6 donors within 24 hours. On the second day after irradiation, groups of mice received 8 × 10^5^ MACS-purified splenic T cells (Pan T Cell Isolation Kit II, Miltenyi Biotec) from C57BL/6 donors to induce GVHD as described previously (47). For the isolation of Tregs, spleen cells were enriched for CD25^+^ cells using the CD25 MicroBead Kit from Miltenyi Biotec and further purified by FACS using CD4 and CD25 antibodies on a MoFlo Legacy Cell Sorter (Cytomation). The purity of Tregs was analyzed by FACS using FoxP3 positivity. Purity was at least 94% in all experiments. Survival and appearance of mice were monitored daily, and the degree of clinical GVHD was assessed every 2–3 days according to Cooke et al. (48) Briefly, animals were scored for 5 clinical parameters graded from 0 to 2 for each criterion: weight loss, posture (hunching), activity, fur texture, and skin integrity. Mice reaching a cumulative GVHD score of 6 or more were sacrificed and counted as deceased in survival analysis.

### Calculation of the efficiency of IgG absorption and serum half-life.

For the calculation of the absorption efficiency, the mean value of peak serum IgG levels of nonirradiated BALB/c mice was defined as 100%, and relative to this value, the absorption efficiency of other groups was calculated. To determine serum IgG half-life, an online available half-life calculator from SydPath (The Pathology Service of St Vincent’s Hospital Sydney, Darlinghurst, Australia; http://www.sydpath.stvincents.com.au) was used.

### Statistics.

All statistics were calculated using Prism Software (Graph-Pad Software). For survival analyses, the log rank test was used. For all pairwise comparisons, the Mann-Whitney U test (2-sided) was applied.

### Study approval.

All experiments were conducted in accordance with German Animal Welfare Act (TierSchG) and approved by the Committee on Ethics of Animal Experiments at the Bavarian Government (Az. 54-2532.1-57/12, Az. 54-2532.2-3/08, and 55.2.2-2532-2-835).

## Author contributions

MS conceived the experiments, performed experiments, analyzed data, and wrote the manuscript; IK, NH, and MBH performed experiments and analyzed data; PH, ME, EU, and FBS analyzed data, gave conceptual advice, and revised the manuscript; WJB gave conceptual advice and wrote the manuscript; and MM and THW conceived the experiments, analyzed data, and wrote the manuscript.

## Supplementary Material

Supplemental data

## Figures and Tables

**Table 1:**
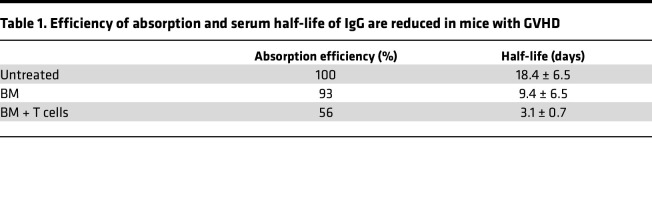
Efficiency of absorption and serum half-life of IgG are reduced in mice with GVHD

## Version 1 01/31/2023

In-Press Preview

## Version 2 03/08/2023

Electronic publication
