# Supplementary material for: Murine cytomegalovirus reactivation concomitant with acute graft-versus-host disease is controlled by antibodies
Source: JCI Insight. 2023 Mar 8;8(5):e149648. doi: 10.1172/jci.insight.149648 (PMC10077468; doi:10.1172/jci.insight.149648)

Suppl. Figure 1

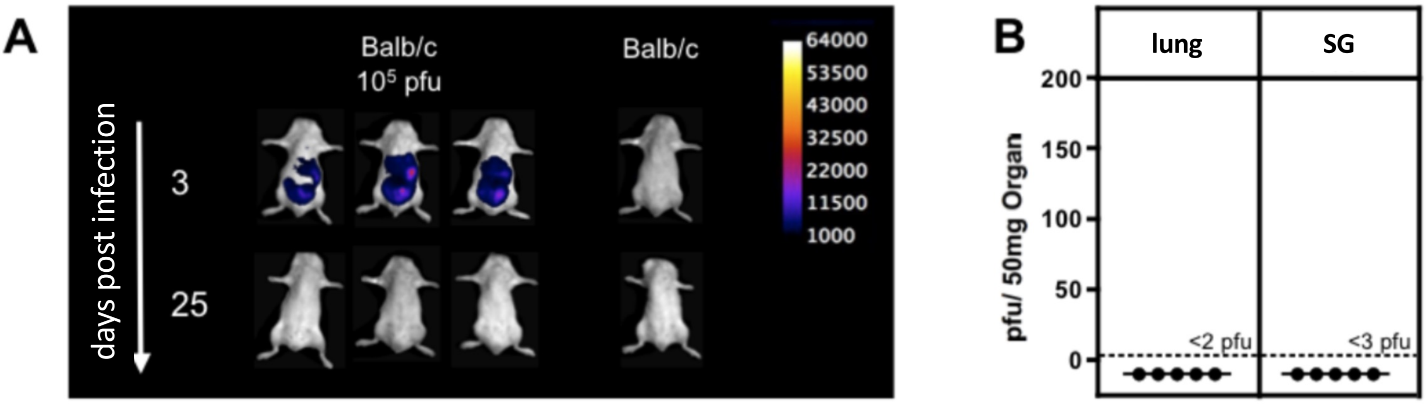

Suppl. Figure 1: Efficient virus control in Balb/c mice after infection with mCMVΔm157-luc. Balb/c mice were infected with 10^5 pfu mCMV**Δ**m157-luc. (A) Representative bioluminescence imaging analysis at d3 and d25 after infection. An uninfected mouse served as control. (B) Quantitative determination of virus load in lung and salivary gland (SG) at day 28 after infection by plaque assay. Dashed line represents lower level of detection.

Suppl. Figure 2

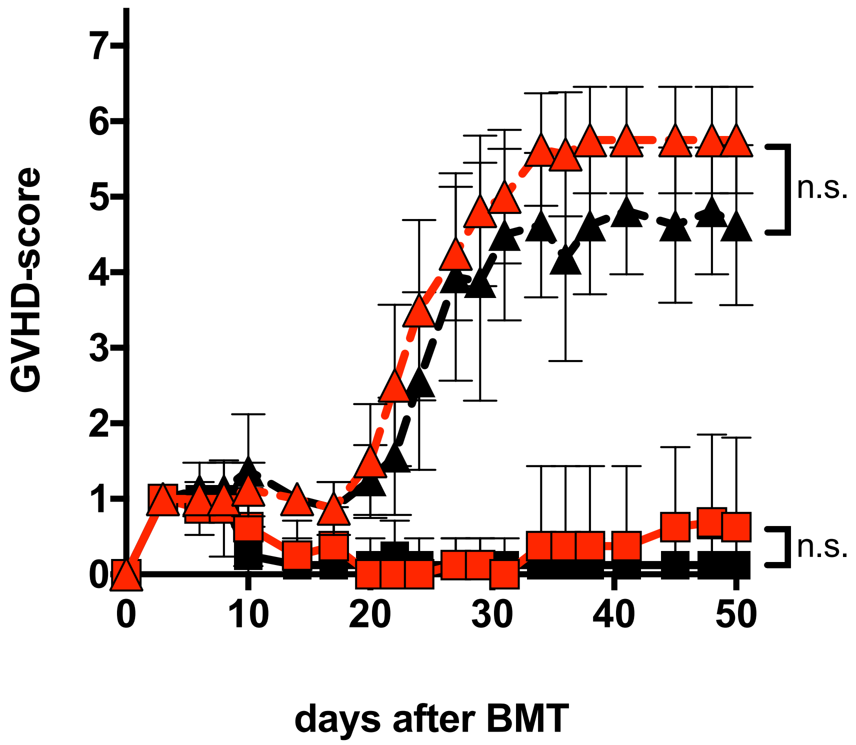

Suppl. Figure 2: Clinical GVHD score for infected and uninfected Balb/c mice
MCMV infected Balb/c mice (red symbols) and uninfected Balb/c mice (black symbols) were lethally irradiated and received 5 x 10^6 T cell-depleted bone marrow cells (BM) with (triangles) or without (squares) 8 x 10^5 T cells from C57BL/6 donors. The mean +/- SD of the GVHD score is presented for groups of 8 mice. P>0.05 for infected versus uninfected mice (2way ANOVA)

Suppl. Figure 3

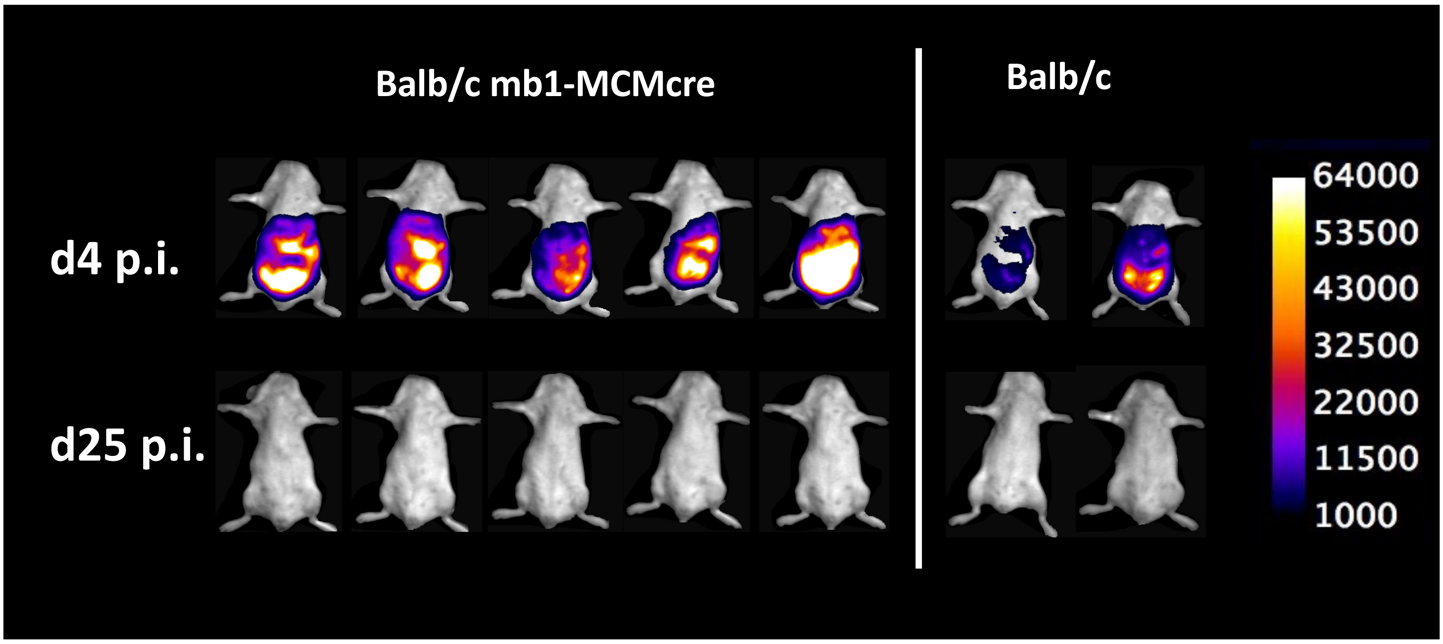

Suppl. Figure 3: Control of MCMV infection in Balb/c B cell deficient mice. 5 representative B-cell deficient Balb/c mice (mb1-MCMcre homozygous knockin mice) are shown in bioluminescence. *In vivo* bioluminescence performed on day 4 and day 25 after infection with 10^5 pfu mCMVΔm157-Luc i.p. Infected wildtype Balb/c mice are shown for comparison.

Suppl. Figure 4

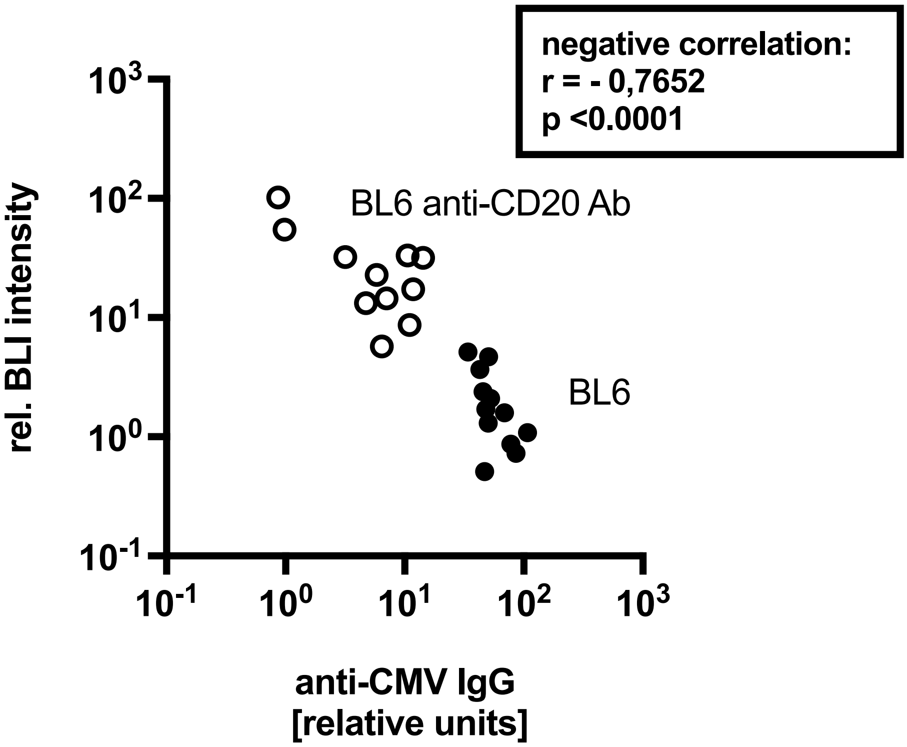

Suppl. Figure 4: Correlation of MCMV viral load and antibody titers day 8 after BMT in C57BL/6 mice and anti-CD20 treated C57BL/6 mice. n=12 animals for each group from one experiment; spearman correlation test.

Suppl. Figure 5

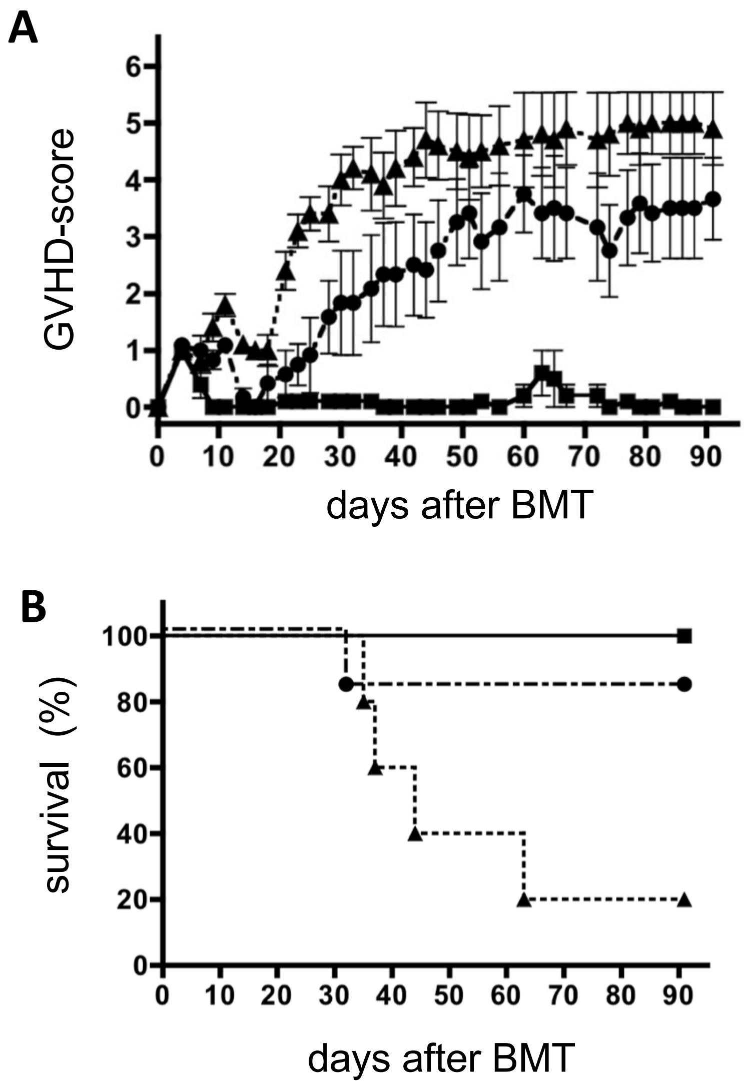

Suppl. Figure 5: Suppression of lethal GVHD in non-infected recipients by transfer of donor Treg cells.
BALB/c mice were lethally irradiated and received 5 x 106 T cell-depleted bone marrow cells only (squares), coinjected with 8 x 10^5 T lymphocytes (triangles) or coinjected with 8 x 105 T lymphocytes and 1.2 x 10^6 CD25+ Treg (circles) from C57BL/6 donors. (A) GVHD index over a period of 91 days post BMT, mean values +/- SEM. (B) Survival curve of infected recipients over a period of 91 days post BMT.

Suppl. Figure 6

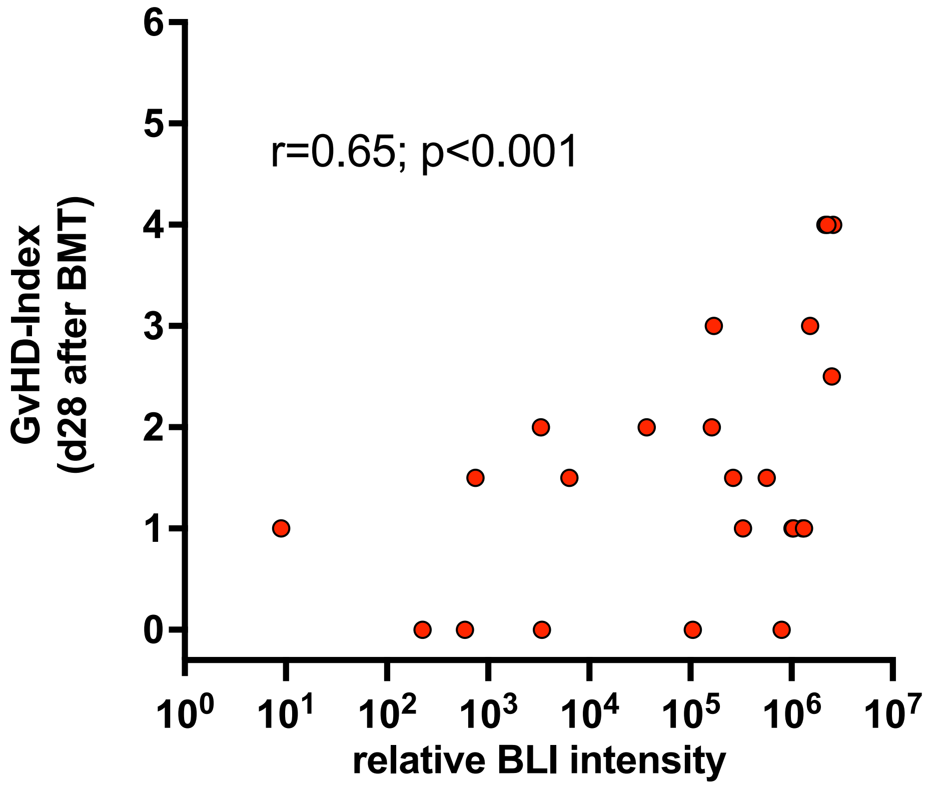

Suppl. Figure 6: Correlation of GVHD index and viral load day 28 after BMT in Treg treated animals only. n=24 animals from 3 independent experiments; spearman correlation test.

Suppl. Figure 7

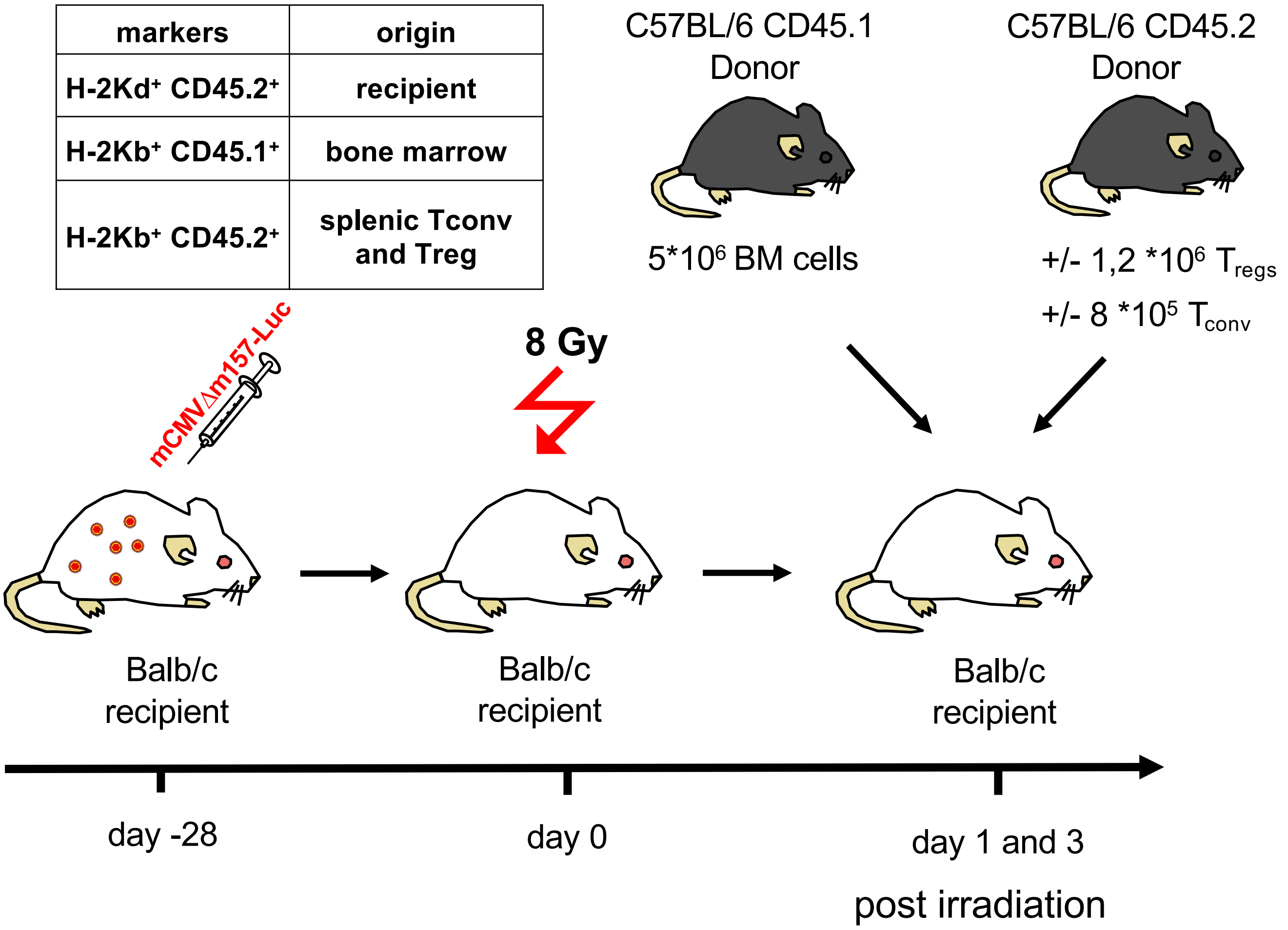

Suppl. Figure 7: Experimental setup for therapeutic T_{reg} transfer with allotype marked donor bone marrow and splenic T cells

Suppl. Figure 8

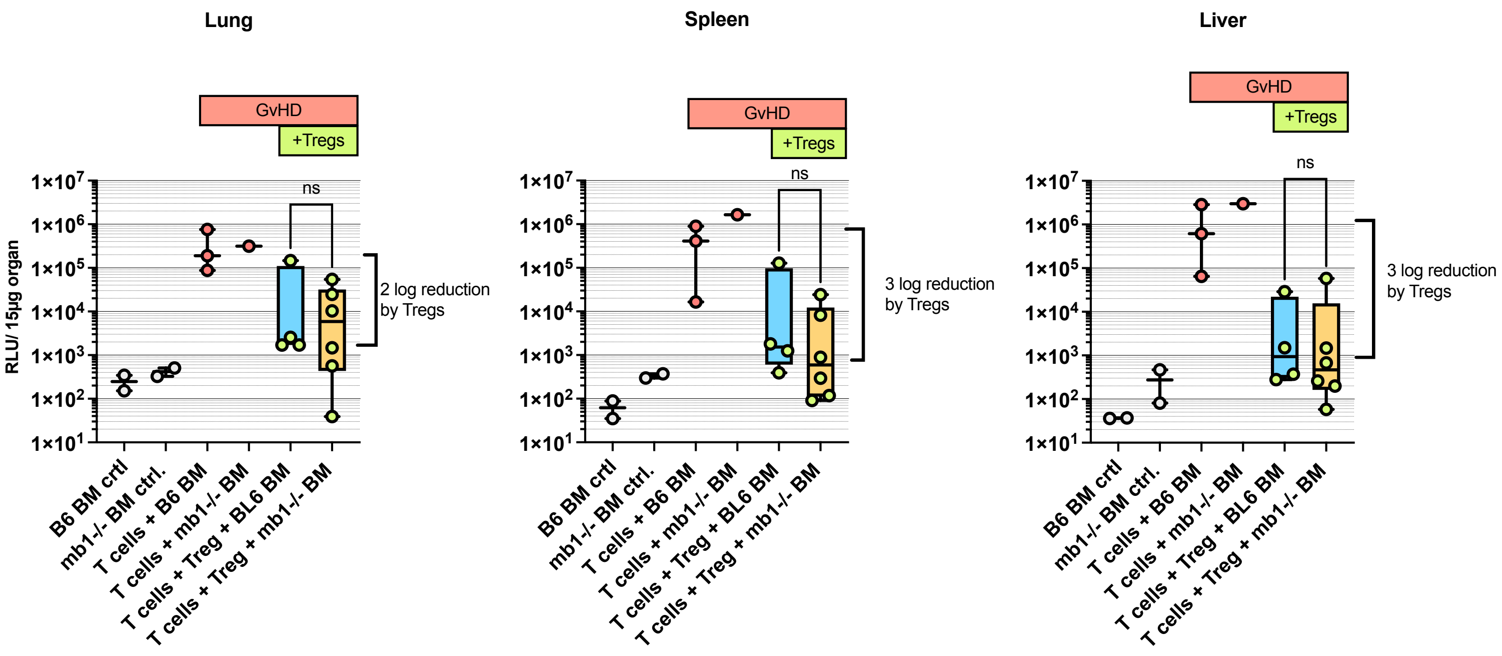

Suppl. Figure 8: Influence of *de novo* bone-marrow derived antibody production on MCMV control after Treg transfer

MCMV infected BALB/c mice were lethally irradiated and received 5 x 10^6 T cell-depleted bone marrow cells (grey symbols) coinjected with 8 x 10^5 T lymphocytes (red symbols) or coinjected with 1.2 x 10^6 $CD25^+$ Treg (green symbols) from C57BL/6 donors. T cell-depleted bone marrow cells were derived from wildtype C57BL/6 or B cell-deficient C57BL/6 mice (denoted as mb1-/- BM). On day 28 after transplantation mice were sacrificed and MCMV infection was quantified as relative luciferase activity in organ lysates. The average reduction of MCMV virus load in experimental groups with Treg transfer is denoted as approximate log-fold reduction. Data are from one experiment with 2 individuals for no-GVHD control (grey symbols), 5 individuals for the GvHD control group (GvHD without Tregs, 2 and 4 mice respectively had to be anesthetized due to severe sickness) and 10 individuals each for the Treg group (6 and 4 mice respectively has to be anesthetized due to severe sickness before reaching the endpoint). ns: not significant

Suppl. Figure 9

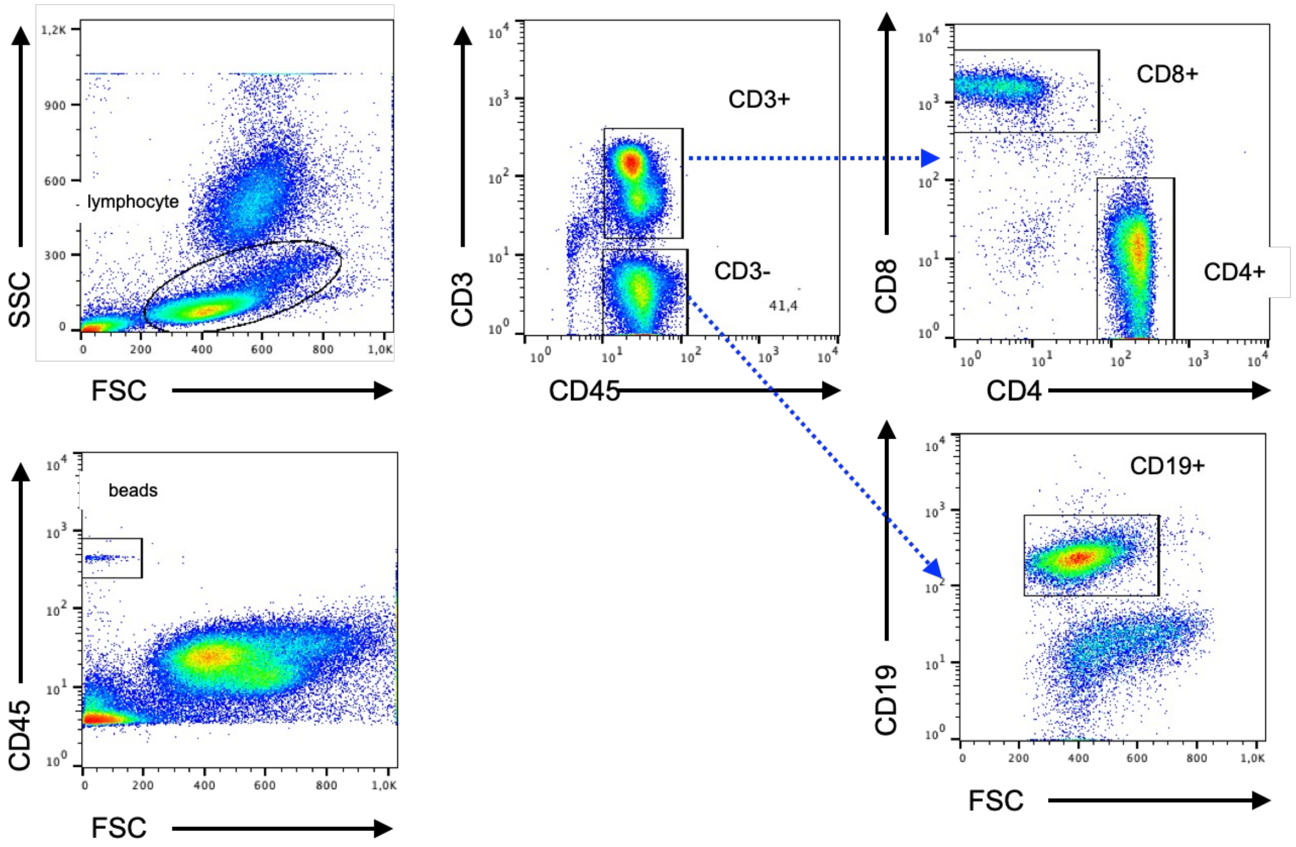

Suppl. Figure 9: Gating strategy for determination of absolute cells numbers of CD19+, CD4+ and CD8+ lymphocytes in peripheral blood. A blood sample from a wildtype Balb/c mouse served for setting the gates. Graded numbers of ProCOUNT counting beads (Becton, Dickinson) were added to the blood samples and beads were detected as FSC low, SSC high, APC high particles (beads gate).

Visual Abstract

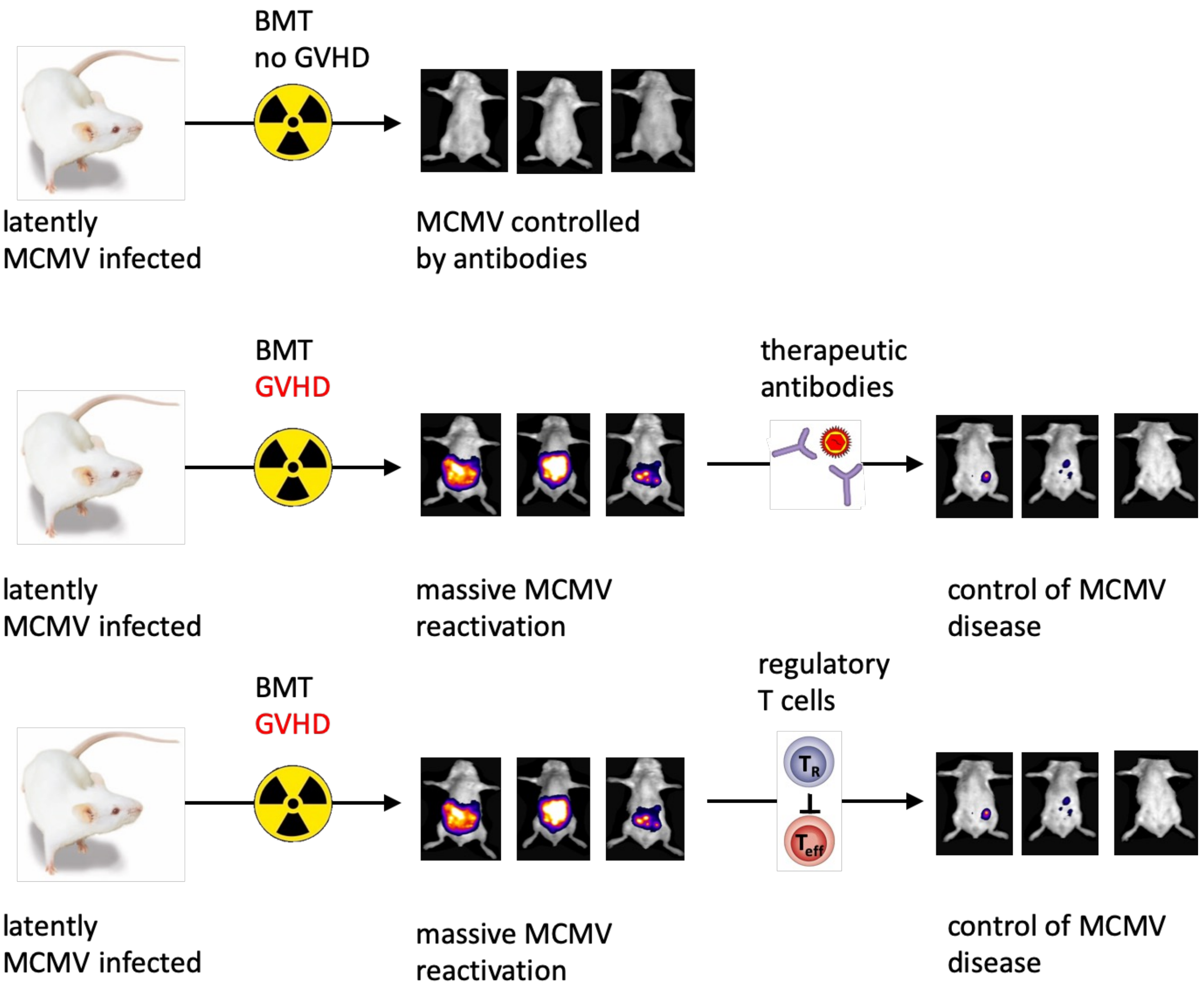

Supplement: Supplemental data [file jciinsight-8-149648-s040.pdf]
